# Underrepresentation of Indigenous mental health professionals in Bangladesh

**DOI:** 10.1192/bji.2024.13

**Published:** 2024-08

**Authors:** Md. Omar Faruk, Miguel R. Ramos, Umay Ching

**Affiliations:** 1 Department of Clinical Psychology, University of Dhaka, Dhaka, Bangladesh.; 2 Department of Psychology, Louisiana State University, Baton Rouge, Louisiana, USA; 3 Department of Social Policy, Sociology and Criminology, University of Birmingham, Birmingham, UK

**Keywords:** Mental health services, Indigenous people, mental health professionals, Bangladesh, underrepresentation

## Abstract

Indigenous people worldwide are at increased risk of mental health problems compared with non-Indigenous people. Longstanding impacts of colonisation, systematic exclusion from rights and subsequent discrimination, and lack of access to quality education and healthcare, including mental healthcare, have been identified as contributory factors to these disproportionate mental health problems. With limited access, Indigenous people are less likely to seek healthcare, owing to the insufficient number of healthcare professionals representing Indigenous communities. In the face of growing numbers of mental health problems in Indigenous people in Bangladesh, this paper sheds light on the inadequate number of mental health professionals, particularly from Indigenous communities, and the potential impacts of this on the well-being of Indigenous people, and considers ways to increase representation of Indigenous mental health professionals. The aim is to ensure that the mental health system in Bangladesh is inclusive and embraces the country's diversity.

In Bangladesh, a low- and middle-income country (LMIC) in the South-Asian region with a population of over 163 million, approximately 18% of adults and 12% of children are reported to have mental health problems.^1^ There are enormous treatment gaps of approximately 92% for adults and 94% for children.^2^ Against this backdrop, the numbers of psychiatrists and psychologists in Bangladesh are 0.16 and 0.007 per 100 000 population, respectively,^1,3^ and the number of nurses working in the mental health sector is 0.873 per 100 000 population.^4^ Similar situations can be found in other low-income countries; evidence suggests that low-income countries have 0.1 psychiatrists and 0.3 psychiatric nurses per 100 000, whereas these numbers are 120 and 75 times higher, respectively, in high-income countries.^5^ For example, the number of mental health professionals in the USA is 105 per 100 000 people, and Canada, Switzerland and Australia have approximately double that ratio.^6^ In addition, the funding allocated for mental healthcare in low-income countries is estimated to be 0.08 USD per person, compared with 0.37 USD in LMICs and 52.73 USD in high-income countries.^7,8^ The inadequate funding in LMICs has been attributed to partial incorporation of mental healthcare into overall healthcare systems,^9^ poor governance and a dearth of mental health professionals.^10^ It has also been argued that poor administration, characterised by neglect of crucial strategies and legal frameworks and a propensity to overlook innovative health system models that could improve mental healthcare, poses a barrier to implementing effective mental healthcare systems in LMICs.^10,11^ In Bangladesh, the annual expenditure for mental healthcare is approximately 0.44% of the total budget.^3^ Overall, mental healthcare is concentrated in the principal cities, where the majority of professionals prefer to work, making it more difficult for people in rural areas to access care,^1^ especially Indigenous people residing in remote hill tract areas in the south-eastern part of Bangladesh.^12^

Historically, Indigenous people worldwide have experienced disproportionate mental health problems compared with non-Indigenous people,^13^ including increased rates of suicide, depression and substance misuse.^14,15^ Nevertheless, Indigenous people are less likely to seek mental healthcare,^16^ largely owing to inaccessibility of mental health professionals, fear and mistrust of a system run by the majority-dominated government, fear of faulty or inflated diagnosis, feelings of shame in seeking care, and discomfort around disclosing delicate issues to a professional without sufficient cultural competency.^17,18^ However, the key factors contributing to elevated mental health repercussions revolve around the impact of colonial hegemony.^13^ Land dispossession, infiltration of dominant cultures, and indifference towards protection of languages and cultural practices are among the assimilatory factors perpetuating widespread mental health problems. Bangladesh, which is home to 54 Indigenous communities with distinct cultural identities, residing in both plain and hill tract areas, is no exception, and serious mental health problems have been reported in its Indigenous people.^12^ In parts of the hill tracts, extreme poverty, lack of quality education, restricted access to healthcare – partly owing to geographical isolation – inadequate transport facilities, and increased militarisation reducing freedom of movement are all specific contextual factors contributing to exacerbated mental health problems. In addition to the higher prevalence of mental health problems, opportunities for accessing mental healthcare are extremely limited. Whereas the number of professionals in Bangladesh is markedly insufficient in general, the representation of psychologists from Indigenous communities, irrespective of discipline, is particularly low. The following section provides insights into the existing gap, potential underlying factors and ways forward to increase representation of mental health professionals from Indigenous communities.

## Reasons for disproportionate representation

The reasons for the disproportionate representation are embedded in socioeconomic structures and longstanding discrimination. As in other parts of the world, Indigenous people in Bangladesh have historically experienced systematic disadvantages such as poverty and restricted access to education and healthcare. The Chittagong Hill Tracts (CHT), a geographical region in south-eastern Bangladesh, is home to the majority of Indigenous people in the country. People in CHT have faced continued poverty that directly reduces their educational opportunities.^19^ The poverty rate is estimated to be high, with 86% of households living below the poverty line.^20^ Educational attainment has constantly been low owing to political and social marginalisation.^19^ For example, a survey showed that 65% of children aged 5–16 years discontinued education for a host of reasons, including not having schools in their local community, not feeling welcome in school and struggling with instructions that are not in their first language.^20^ Indigenous people in CHT also experience inadequate and inaccessible healthcare^21,22^ owing to lack of knowledge about healthcare needs, geographical dispersion, elevated medical expenses, traditional health practices, lack of reliance and trust, language barriers and unhelpful behaviour of healthcare personnel.^21^

There is also limited access to quality education and training programmes in mental health in the appropriate languages in Indigenous regions. Furthermore, geographic isolation, financial hardships and the lack of a culturally sensitive curriculum impede educational opportunities in Indigenous communities. These sustained disparities may have resulted in the insignificant numbers of Indigenous mental health professionals in the field of psychology.

The underrepresentation of Indigenous mental health professionals is reflected in a cycle of limited mentorship, role models and support networks, all of which are responsible for perpetuating the underrepresentation. For example, the absence of representation discourages aspiring Indigenous people from pursuing careers in psychology. In addition, insufficient resources and funding devoted to mental health services in Indigenous communities affect the demand for Indigenous mental health professionals. As previously noted, Bangladesh spends approximately 0.44% of its total health budget on mental health expenditure.^1,3^ The lion's share of the allocated funds (approximately 67%) is devoted to mental hospitals in principal cities.^1^ This allocation fails to prioritise the mental health and education of Indigenous people residing in remote hill tract areas. Moreover, Indigenous communities may prioritise other pressing needs over mental health services. For example, individuals in such communities in Bangladesh prefer to have government employment, as this enables them to gain social status, recognition and security, which the mental health profession is unable to ensure.

The reasons may also include mental illness stigma in Indigenous communities,^23^ which can discourage people from choosing careers in mental health. In Indigenous communities in Bangladesh, mental illness is believed to be caused by evil spirits, and this prevents people from seeking care.^24^ Therefore, people resort to traditional healing practices (e.g. faith healing and rituals to fend off evil spirits) to be ‘cured.’ These cultural practices conflict with Western approaches, which are primarily based on values reflected in the ‘WEIRD’ psychology that characterises ‘Western, Educated, Industrialized, Rich and Democratic’ countries.^25^ Psychological understanding has historically been dominated by these countries, with little regard for cultural and racial diversity.^26^ Therefore, it is reasonable to assume that there may be a different understanding of mental health and care in Indigenous communities, with a conceptualisation of psychopathology that is distinct from that of WEIRD psychology. Mental healthcare in Bangladesh is largely dominated by WEIRD values, placing little value on cultural narratives such as the development of psychopathology and coping strategies, or on healing processes (i.e. faith healing). This is particularly crucial for Indigenous people, who have inadequate representation as key stakeholders in designing mental healthcare systems with their knowledge, understanding and wisdom. The lack of incorporation of such factors further promotes epistemic injustice, which may eventually result in lack of treatment adherence – an issue frequently evidenced in scholarly works.^15–17^

Finally, a perceived lack of cultural competency among non-Indigenous mental health professionals in dealing with Indigenous people with mental health complaints leads to a sense of mistrust and dissatisfaction, further discouraging Indigenous people from entering the field. Structural barriers such as healthcare policies and institutional biases also contribute to the underrepresentation of Indigenous mental health professionals.

## Strategies to increase representation

A multimodal strategy is needed to address the abovementioned issues. Here, we provide a host of recommendations to increase the number of Indigenous mental health professionals in Bangladesh.

Task sharing, an approach to expand mental healthcare in resource-limited settings, especially in LMICs, could be a viable solution to the existing crisis. This approach emphasises transferring mental healthcare skills from experts to non-experts through continued training and supervision. Skills needed to identify common mental health disorders and provide primary care are at the heart of task sharing. Research has demonstrated the promise of this approach in providing mental healthcare in resource-limited contexts.^27^ It complements efforts in restructuring Western healthcare models that have recognised the importance of community participation and intercultural healthcare provisions with a view to reducing inequality.^28^ When designing and implementing a task-sharing approach, it is imperative to take into account Indigenous people's representation and the cultural competency of both Indigenous and non-Indigenous people. It is also important to facilitate exchange of professional competencies between Indigenous and non-Indigenous mental health professionals, emphasising Indigenous perspectives, histories and practices. This may help to overcome problems posed by geographical distance, unavailability of resources and infrastructure, lack of community engagement and misconceptions, all of which are strong barriers to effective healthcare.^29^ Ensuring mental healthcare through task sharing has the potential to draw the attention of Indigenous students and stakeholders to the field of mental health. Such endeavours could contribute to reducing the disparities in Bangladesh's healthcare system.

Having distinct dialects, Indigenous people in Bangladesh experience language barriers in communication with non-Indigenous people speaking Bangla, the state language. Disparities can be perpetuated when individuals do not have mastery over the dominant language or mental health literacy in their native language. To address this challenge, it would be helpful to have a curriculum focusing on bilingual and bicultural education and learning. In Bangladesh, research testing this type of curriculum has shown significant improvements in children's language development, quantitative reasoning, and educational and cultural awareness. Besides, children trained using a curriculum characterised by active participation showed more engagement.^19^ In Canada, language-in-education policies are enforced with continued financial allocation to increase numbers of Indigenous language speakers, respecting cultural safety, dignity and the right of individuals to be educated in their native language, and adoption of culturally meaningful curricula.^30^ Scaling up the bilingual and bicultural education curriculum with components of mental health could impart literacy among Indigenous people, which in turn may contribute to an increased workforce in the future. This could also help to revitalise Indigenous languages that are on the verge of extinction.^30^

Reforming the education system could gradually help to reduce disparities. For example, psychology departments in educational institutions across the country could offer scholarships, grants and financial aid to Indigenous students interested in building careers in psychology. Allocating more resources could benefit Indigenous students pursuing careers in this discipline. Besides, a mentor programme focusing on guidance, support and network opportunities could be useful in engaging Indigenous students. Finally, offering detailed career pathways and strategies necessary for a stable job might attract Indigenous people to the mental health profession.^31^

Mass awareness campaigns targeting Indigenous communities and focusing on mental health problems and care, including the importance of Indigenous mental health professionals, may help to reduce the gap posed by the current disproportionate representation of mental health professionals. Expansion of access to education, training and research will serve as a catalyst to increase the representation of Indigenous mental health professionals. It is crucial to advocate for policies that support diversity and inclusion in the mental health workforce.

Cultural competency training for mental health professionals to enable them to better understand and respect Indigenous people's cultures might defuse hesitancy in seeking care due to a lack of cultural humility.^32,33^ Having cultural competency also offers a supportive work environment, which might be considered useful to those choosing a career in psychology.^31^

There should be continued collaboration with organisations and institutions led by Indigenous people, with the aim of creating opportunities for education and training (e.g. internships). Organisations working on mental healthcare should support the development of Indigenous-focused mental health programmes. Finally, adopting policies that recognise and support the rights of Indigenous people in the healthcare system could contribute to increasing their representation in the mental health profession.^34^

## Conclusion

Addressing the underrepresentation of Indigenous mental health professionals across the world, including in Bangladesh, is crucial to ensure that our efforts to provide mental healthcare for all are culturally responsive and equitable. In addition, this practice will stimulate trust within Indigenous communities, reduce disparities, and preserve and promote cultural practices. Therefore, increasing representation in the mental health profession warrants a long-term commitment, involving Indigenous communities at every stage and focusing on sustained efforts and resources, reflecting the core essence of the saying ‘nothing about us without us’.^35^ Ongoing evaluation, adaptation and formulation of strategies will be necessary to sustain efforts to increase Indigenous people's stakes in the mental health profession.
